# Effective Part-Task Training as Evidence of Distinct Adaptive Processes with Different Time Scales

**DOI:** 10.1371/journal.pone.0060196

**Published:** 2013-03-27

**Authors:** Sandra Sülzenbrück, Herbert Heuer

**Affiliations:** Leibniz Research Centre for Working Environment and Human Factors, Dortmund, Germany; VU University Amsterdam, The Netherlands

## Abstract

For some types of visuo-motor transformations like large visuo-motor rotations or the complex transformation of a sliding first-order lever, distinct adaptive processes have been hypothesized that produce a rapid, discrete approximation of the transformation and a slow, graded fine tuning, respectively. Here we investigate whether part-task training of only the second of these processes, namely the fine tuning, transfers to the subsequent performance in a condition with the full transformation of the sliding first-order lever. Therefore, we compared performance of three groups with different practice conditions during transfer to the full transformation. While two groups only practiced the fine tuning without the right-left inversion of the lever prior to transfer, a third group practiced the full lever transformation. Our results show a positive, but less than perfect transfer of the isolated practice of the fine tuning on performance with the full transformation. For the fine tuning itself, transfer was not reliably different from being perfect. The observation that the fine tuning can be acquired separately and added to the later adaptation to the left-right inversion of the lever supports the notion that these slow and fast processes progress rather independently. The additional finding that the preceding acquisition of the fine tuning also facilitates the subsequent rapid process could be due to generalized learning-to-learn or to a more precise assignment of movement errors to the process from which they originate.

## Introduction

Humans are remarkably proficient in tool use. This proficiency is based on adaptation to transformations of bodily movements into movements of the effective parts of the tools. Models of the mechanisms involved posit that distinct processes contribute to adaptation to sensori-motor transformations [1]–[5]. Such processes are claimed to differ in various characteristics such as their dynamics and their accessibility to cognitive control. Here we test a particular two-process hypothesis for a complex kinematic transformation by means of a transfer study.

For some types of kinematic transformation two distinct processes have been suggested that differ both in their rate and in the type of changes they produce, namely discrete or graded ones. For example, according to Abeele and Bock [6] adaptation to large visuomotor rotations between 90° and 180° invokes a fast process which produces a discrete point-symmetric approximation, that is, an abrupt change of the arm-movement direction by 180° relative to the target direction. The slow process then produces a graded shift to the smaller correct rotation. A similar combination of discrete and continuous processes has been hypothesized for adaptation to right-left reversal [7] and for the acquisition of the internal model [8]–[9] of the complex kinematic transformation of a sliding first-order lever [10]–[12]. At first glance the transformation of a sliding first-order lever may appear as a somewhat esoteric object of study. However, this is the type of transformation of typical instruments used in minimal access surgery [13]–[15], and its mastery is crucial for the clinical outcome [16].

The mastery of a tool implies the production of an input (movements of the hand) appropriate for the desired output (correct movement of the effective part of the tool). Determination of the appropriate input requires an inversion of the respective visuo-motor transformation [17]. The inversion can be approximated in two different ways [18], either under visual closed-loop (or feed-back) control or under visual open-loop (or feed-forward) control. When concurrent visual feedback is available, movement corrections can occur on-line in the course of the movements, generally resulting in accurate movements if there is no time pressure. Successful closed-loop control does not require the acquisition of a sufficiently accurate internal representation or internal model relating hand movements to the movements of the effective part of the tool [19]. In contrast, open-loop (or feed-forward) control requires such an internal model of the visuo-motor transformation. With a correct internal model, the input required to reach a desired output can be planned before the movement is started. Therefore, the acquisition of an internal model of a visuo-motor transformation enables accurate movements without concurrent visual feedback.

Control of a sliding first-order lever relies both on visual feedback and on an acquired internal model of the transformation. The characteristics of the internal model of the transformation of the tool can be assessed when visual feedback is turned off [8], [20]–[22]. Based on performance in visual open-loop trials, a rapid and a slow process have been claimed to contribute to the acquisition of the internal model [10]–[12]. The rapid process should result in a discrete line-symmetric approximation of the transformation, whereas the slow process should consist of a graded fine tuning of the internal model. These two processes will be described in more detail.

The first process is rapid and produces a line-symmetric approximation of the kinematic transformation of the sliding lever: hand movements come to end in positions that are laterally symmetric to the visually presented targets. The symmetry axis is the vertical or horizontal axis of the frame of reference with its origin in the start positions of a cursor, representing the position of the end effector on the monitor, and the frame of reference with its origin in the start position of the hand. This approximation accounts for the left-right inversion of lateral movements of the tip of the lever relative to those of the hand, the so-called fulcrum effect [13]. The approximation neglects variations of movement directions (and amplitudes) resulting from a direction-dependent gain factor (gain anisotropy) that depends on the relative lengths of the effort arm and the load arm [23]. The fine tuning of the internal model, which takes this gain anisotropy into account, is hypothesized to be a second and slow process. It gives rise to a gradual shift of the end positions of hand movements away from the line-symmetric approximation to the correct positions.

A sliding first-order lever is a physical object with variable inertial resistance. Its dynamic transformation presents as an inertial anisotropy, which means that the inertial load depends on the direction of movement in a way that is again position-dependent [23]. For translations of the lever (sliding forward and backward), the inertial load is constant, namely the mass of the lever. In contrast, for rotations of the lever there is a different inertial load which depends on the relative lengths of the effort arm and the load arm. The inertial load is relatively smaller, the longer the effort arm is, and it is relatively higher, the longer the load arm is. For all directions that are combinations of rotations and translations, the inertial load is a combination of the pure cases. The inertial anisotropy has the effect that the direction of movement deviates from the direction of force.

The hypothesis of distinct processes of sensori-motor adaptation suggests that the acquired internal model of a transformation can be decomposed into different components that can be acquired separately. Positive transfer of previously learned components of a transformation on subsequent performance has been shown, for example, for dynamic and kinematic transformations [24]–[25] and for different dynamic transformations [26].

In the case of the sliding first-order lever the hypothesized distinct processes are fast and slow. Their outcomes, the line-symmetric approximation and the fine tuning, can be observed in sequence, and it is unknown whether the processes also start in sequence or concurrently. The assumption of distinct processes of sensori-motor adaptation implies the hypothesis that the order of fast and slow processes can be reversed, even when the processes operate in sequence (except when the output of the fast process serves as an input for the slow process). This hypothesis is tested in the present study for the acquisition of an internal model of the complex transformation of a sliding first-order lever.

Specifically we ask whether part-task training of the slow fine tuning transfers to the acquisition of the full transformation of the lever. Therefore the mechanical transformation of a sliding first-order lever was practiced by one group of participants (group *inverting physical lever*). For two other groups we removed the feature of the kinematic transformation associated with the rapid discrete approximation; in consequence these participants only practiced the fine tuning. To investigate the impact of the dynamic transformation, only one of these groups used the physical lever (group *non-inverting physical lever*), while the other group used a pen with a constant mass and therefore a constant inertia (group *non-inverting virtual lever*). All groups were transferred to the full lever transformation after practice, and performance differences depending on the type of prior practice were analyzed.

## Methods

### Ethics statement

All participants had given written informed consent prior to the start of the experiment. The experiment was conducted at the Leibniz Research Centre for Working Environment and Human Factors in Dortmund, Germany, in accordance with the ethical standards laid down in the 1964 Declaration of Helsinki. Behavioral studies like these which do not put any load on the participants are approved by the ethics committee of the Leibniz Research Centre for Working Environment and Human Factors without requiring an individual request for approval.

### Participants

Sixty right-handed volunteers with normal or corrected-to-normal vision participated in this study (mean age 24.1±3.32 years, 30 female). Participants were assigned to three groups with different practice conditions (10 female participants per group), group *inverting physical lever*, group *non-inverting physical lever*, and group *non-inverting virtual lever*. All participants received 22 € for taking part in the experiment.

### Apparatus

**Figure 1:**
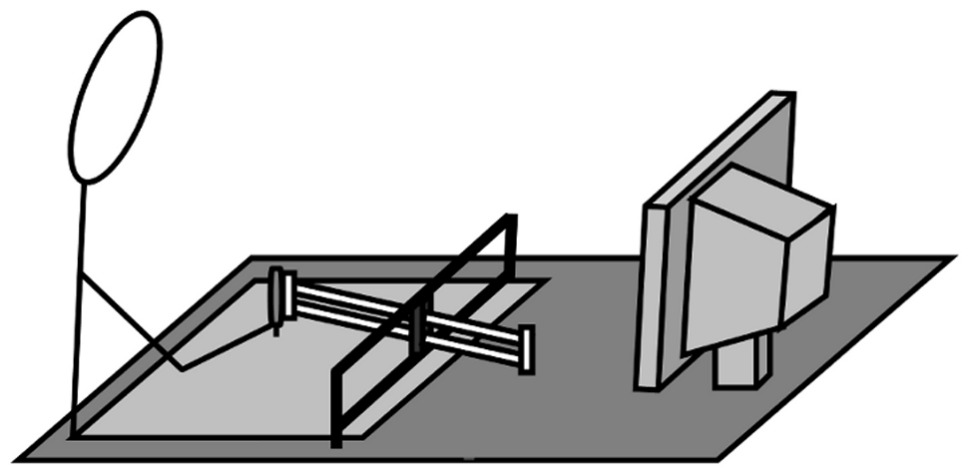
Experimental setup for the physical and the virtual lever.

Figure 1 illustrates the experimental setup. Participants sat in front of a 17-inch computer monitor with a resolution of 1280 pixels x 1024 pixels and a refresh rate of 60 Hz which was placed on a table. A Wacom digitizer with a resolution of 1280 pixels x 1024 pixels and an active area of 324 mm x 243 mm (Wacom Europe GmbH, Krefeld, Germany) was located on the table between the participant and the monitor. At the far edge of the digitizer a vertical frame was mounted that carried the bearings of a 355 mm long sliding horizontal first-order lever. The mass of this lever was 0.37 kg and its moment of inertia was 0.0061+0.37 r^2^kgm^2^, where r is the distance of the center of mass of the lever from the fulcrum. Friction was minimized for rotations around the vertical axis (no movement was possible around the horizontal axis) and for translations through appropriate ball-bearings. In conditions where the physical lever was used, a pen was attached to its proximal end. When the virtual lever was used and during trials without a tool (familiarization and pre-test), the pen was dismounted from the lever. In all trials the position of the pen (x-y-coordinates) on the digitizer was recorded with a sampling rate of 60 Hz. An opaque screen 240 mm above the table surface prevented the participants from seeing their hand as well as the apparatus.

### Task

**Figure 2:**
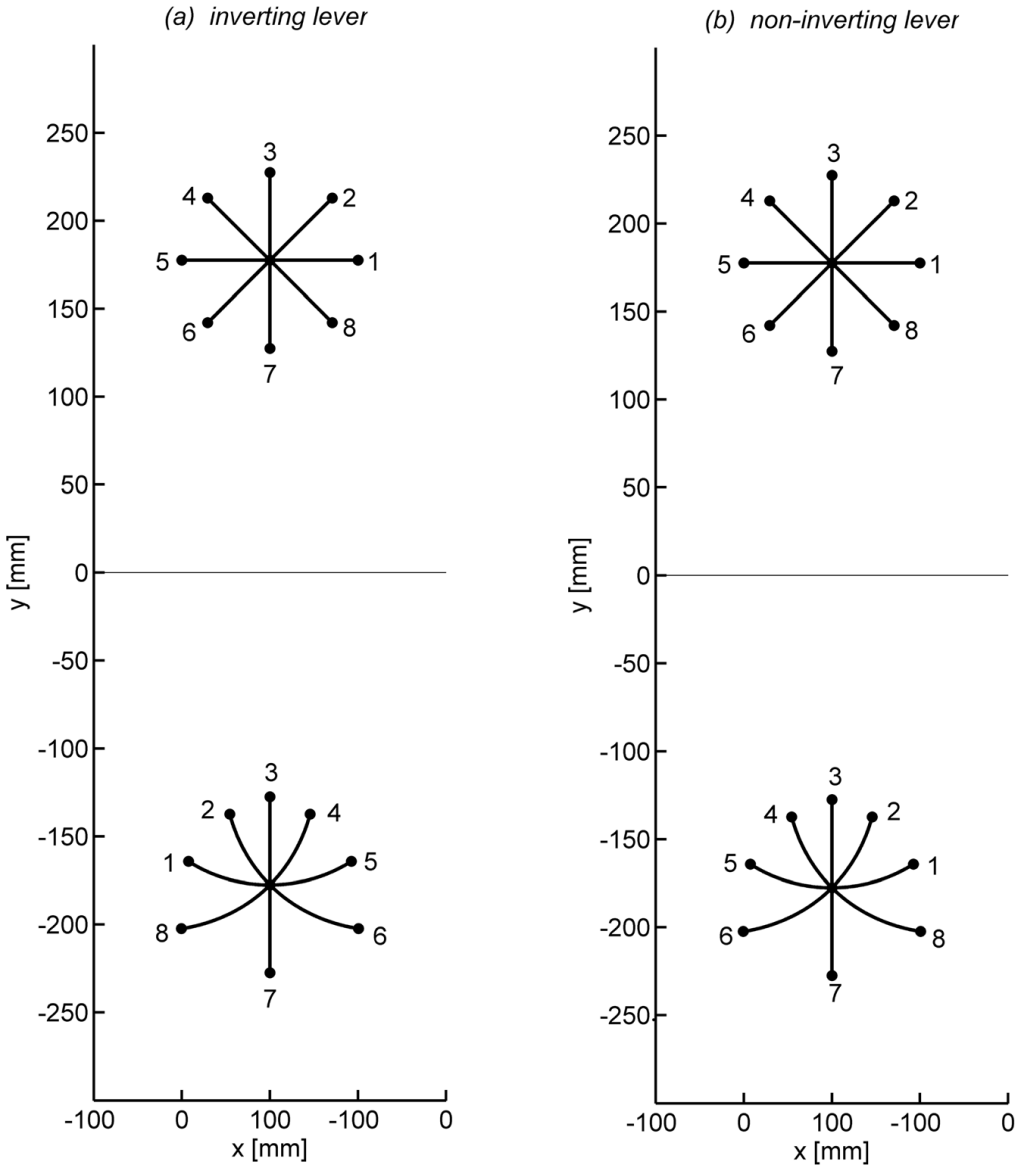
Target configurations with straight paths of the cursor and associated paths of the hand with the inverting lever (a) and the non-inverting lever (b).

Participants grasped a hold mounted at the tip of the pen with two or three fingers of their dominant right hand. They were asked to move the distal tip of the lever as accurately and rapidly as possible to one of eight possible target locations. The position of the distal tip of the lever was represented on the monitor by the position of a cursor (red filled circle of 1 mm radius). The target locations were arranged on a circle of 50 mm radius around a start position (see Figure 2), which was marked by a filled white circle of 1.8 mm radius. Target directions were 0° (to the right), 45°, 90° (forward/upward), 135°, 180°, 225°, 270°, and 315°.

At the beginning of a trial the cursor was invisible. To reach the start position, the participants had to move the pen according to arrows appearing at the edges of the monitor. When the position of the (invisible) cursor had reached a circular region of 10 mm radius around the start position, the cursor appeared on the monitor to assist in homing-in. When the start position had been reached with a tolerance of 1.2 mm and the cursor had remained within the tolerance for 1 s, the color of the start circle changed from white to green. At the same time, a target, a filled white circle of 1.8 mm radius, appeared. This was the signal for the participants to move the distal end of the lever and thus the red cursor as accurately and rapidly as possible to the target position.

Movements were performed under three conditions of visual feedback. In trials with continuous visual feedback the cursor remained visible throughout each movement. The end of the movement was determined by a velocity criterion – velocity had to be less than 5 mm/s for 0.5 s – and an accuracy criterion – the deviation of the cursor from the target had to be less than 1.2 mm. In trials with terminal visual feedback, the cursor disappeared at the start of the movement and re-appeared after its end had been determined by the velocity criterion. The cursor, together with the target, remained visible in its final position for 1 s. Trials without visual feedback were identical except that the cursor did not re-appear after the end of the movement. Only in trials with continuous visual feedback visual closed-loop control was possible, but not in trials with terminal or no visual feedback.

During the experiment the participants encountered different transformations. During familiarization and a pre-test there was a direct 1∶1 mapping, so that the motion of the cursor corresponded directly to the movement of the hand. In the transfer phase all groups used the physical lever. Its transformation is illustrated in Figure 2a by way of showing the hand movements appropriate for straight cursor motions to the 8 targets. During the practice phase group *inverting physical lever* used the normal physical lever as in the transfer phase, but for the other two groups the kinematic transformation was simplified as illustrated in Figure 2b. For these groups lateral hand movements resulted in cursor motions in the same rather than in opposite directions, that is, the lever was not inverting. In group *non-inverting physical lever* the dynamic transformation remained that of the lever, but in group *non-inverting virtual lever* the dynamic transformation was absent. The pen was dismounted from the lever so that the inertia of the tool was no longer anisotropic, but constant.

### Design

Table 1 summarizes the experimental protocol. The experiment began with a block of familiarization trials and a pre-test. The familiarization block consisted of 26 trials with continuous visual feedback. Each of the 8 targets was presented three times in a random order, following two initial warm-up trials. The pre-test consisted of 50 trials without visual feedback. In this test not only each of the 8 targets was presented three times, but there were 8 additional targets which corresponded to the correct end positions of the hand movements in the presence of the transformation. The pre-test served to compare the initial performance levels of the three experimental groups, which could be different just by chance. During familiarization and pre-test, the pen was dismounted from the lever apparatus. In these trials, the visuo-motor transformation was 1∶1, with hand movements resulting in cursor movements of the same direction and amplitude (except for the transformation from the horizontal movement plane of the digitizer to the vertical plane of the computer screen).

**Table 1:** Experimental design.

phase	blocks	transformation			visual feedback
*familiarization*	1	1∶1			continuous
*pre-test*	1	1∶1			no
*practice*	1	inverting physical lever	non-inverting physical lever	non-inverting virtual lever	continuous
	2				terminal
	3				
	4				
	5				
	6				
	7				
	8				
	9				
	10				
	11				no
	12				continuous
*transfer*	1	inverting physical lever			continuous
	2				terminal
	3				
	4				
	5				no
	6				continuous

The pre-test was followed by the practice phase. In total there were 12 blocks of 26 trials each in this phase. In each block each of the 8 targets was presented three times in a random order, preceded by 2 warm-up trials. In the first and last practice block visual feedback was presented continuously during each movement. In the remaining practice blocks visual feedback was terminal, that is, the final position of the cursor was presented together with the target position after the end of the movement. Terminal-feedback practice has been shown to facilitate the fine tuning of the internal model of the transformation of the sliding lever as compared with continuous visual feedback [21]. Only in the block immediately preceding the last block with continuous visual feedback there was no visual feedback at all. Thus, in this block trial-to-trial corrections based on terminal feedback were not possible. Such guidance by knowledge of results presented after each movement can affect performance in principle [27].

Finally, there was a transfer phase in which all three groups of participants used the physical lever. It consisted of 6 blocks of 26 trials each. Again in the first as well as the last block continuous visual feedback was provided. In the intermediate blocks, except for the last of them without visual feedback, terminal visual feedback was presented after each movement.

### Data analysis

The x-y coordinates of the pen (input of the kinematic transformation) as well as the computed x-y coordinates of the tip of the inverting or non-inverting physical or virtual lever (output of the kinematic transformation) were recorded in a Cartesian coordinate system with its origin in the fulcrum. For each movement, each of the four time series was low-pass filtered (fourth-order Butterworth, 10 Hz, dual-pass) and differentiated (two-point central difference algorithm). Start and end of each movement were determined from the tangential velocity of the hand. Beginning at peak velocity and scanning in forward and backward direction, those times were identified at which velocity became smaller than 4 mm/s and remained so for 450 ms. For the computation of averaged movement paths, movements were normalized to a standard duration.

For each trial a number of dependent variables were computed that served both the screening of the data and their statistical analysis. Neglecting the initial familiarization block and the warming-up trials, each participant performed 480 movements. Movements that satisfied one of the following criteria were classified as invalid: (1) trial not finished within 30 s or recording failure; (2) movement time less than 200 ms; (3) movement time longer than 5000 ms (except for movements with continuous visual feedback); (4) path length longer than five times the distance between initial and final position (except for movements with continuous visual feedback); (5) more than 2.5 mm distance of the initial position from the start position (a rare event that occurs when the main movement is preceded by a short initial one). The mean numbers of discarded trials were 4.5, 3.3, and 3.3 (corresponding to 0.9, 0.7, and 0.7%) in the three groups *inverting physical lever*, *non-inverting physical lever*, and *non-inverting virtual lever*, respectively. According to a one-way ANOVA the variation across groups was not significant, *F*(2,57) = 1.0, *p*>.20.

For the remaining trials movement time and Euclidean error of the end position of cursor motion were determined. For each block of trials these were averaged across trials in which the same target was presented, and these means were again averaged across the eight target directions. For trials with terminal or no visual feedback the performance variable was the (Euclidean) error, which captures both the systematic and variable errors of cursor position. For trials with continuous visual feedback accuracy was essentially perfect and errors were zero. As marker of the efficiency of control and thus as performance variable we used movement time.

**Figure 3:**
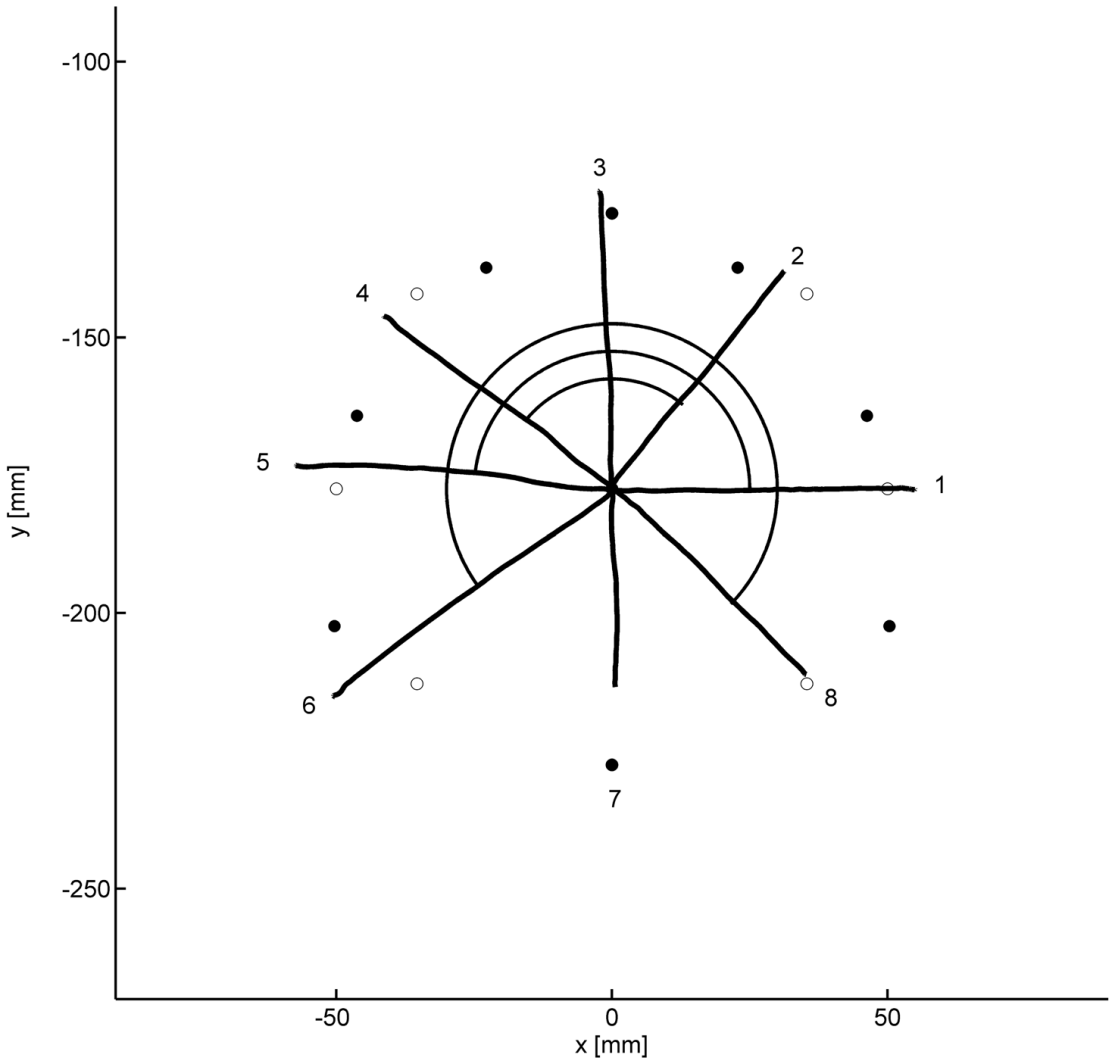
Mean hand-movement paths of a participant of group *non-inverting virtual lever* in the last practice block without visual feedback. Filled circles mark correct endpoints, open circles endpoints appropriate for the symmetry approximation. Angles measured between movements to three pairs of targets are illustrated. For the symmetry approximation these angles are 90°, 180°, and 270°, respectively; for correct endpoints they are smaller by 33.4° on average

For trials without visual feedback, we computed two more dependent variables from the final hand positions that served as markers for the two hypothesized processes, one assessing the compensation of the left-right inversion (line-symmetric approximation) and the other assessing the fine tuning. As a measure for the inversion we computed the percentage of hand movements that ended in the correct quadrant of a rotated coordinate system, that is, the percentage of hand movements with a directional error less than 45°. The rationale and computation of the dependent variable for the assessment of the fine tuning is illustrated in Figure 3. In this Figure the filled circles show the correct end positions of hand movements, and the open circles show the end positions appropriate for the symmetry approximation. In addition averaged movement paths of a single participant of group *non-inverting virtual lever* in the last practice block without visual feedback are shown, numbered according to the targets (cf. Figure 2).

When hand movements ended at the positions appropriate for the symmetry approximation, the angles between movement paths 2 and 4, 1 and 5, and 6 and 8 were 90°, 180°, and 270°, respectively. When the movements ended at the correct positions, these angles were smaller by 30.9°, 32.1°, and 37.3°, that is, by 33.4° on the average. We computed the average angular deviation from the symmetry approximation for each participant and each block of trials from the mean directions of the movements to each target. This measure of the quality of fine tuning, which is 0 for the symmetry approximation and 33.4° for perfect fine tuning, is independent of eventual overall rotations of movement directions relative to target directions. For the computation of the angular deviation only movements were analyzed which compensated for the left-right inversion.

## Results

First, we present baseline performance and illustrate some fundamental characteristics of movements with the sliding first-order lever. Thereafter we report the performance variations during the practice phase and the analysis of transfer performance. Finally we turn to the markers of the two hypothesized processes of learning the transformation, the inversion and the fine tuning.

### Baseline performance

We compared the initial performance level of the three groups of participants in terms of movement time and accuracy in the visual open-loop pre-test. When assessed under identical conditions, performance turned out to be equivalent. According to one-way ANOVAs, neither the variation of movement time, *F*(2,57) = 1.2, *p*>.20, nor of Euclidean errors, *F*(2,57) = 1.0, *p*>.20, was statistically significant. Mean movement times were 1507, 1293, and 1401 ms in the three groups *inverting physical lever*, *non-inverting physical lever*, and *non-inverting virtual lever*, respectively, and the mean Euclidean errors were 19.4, 16.0, and 20.0 mm.

### Representative trajectories

**Figure 4:**
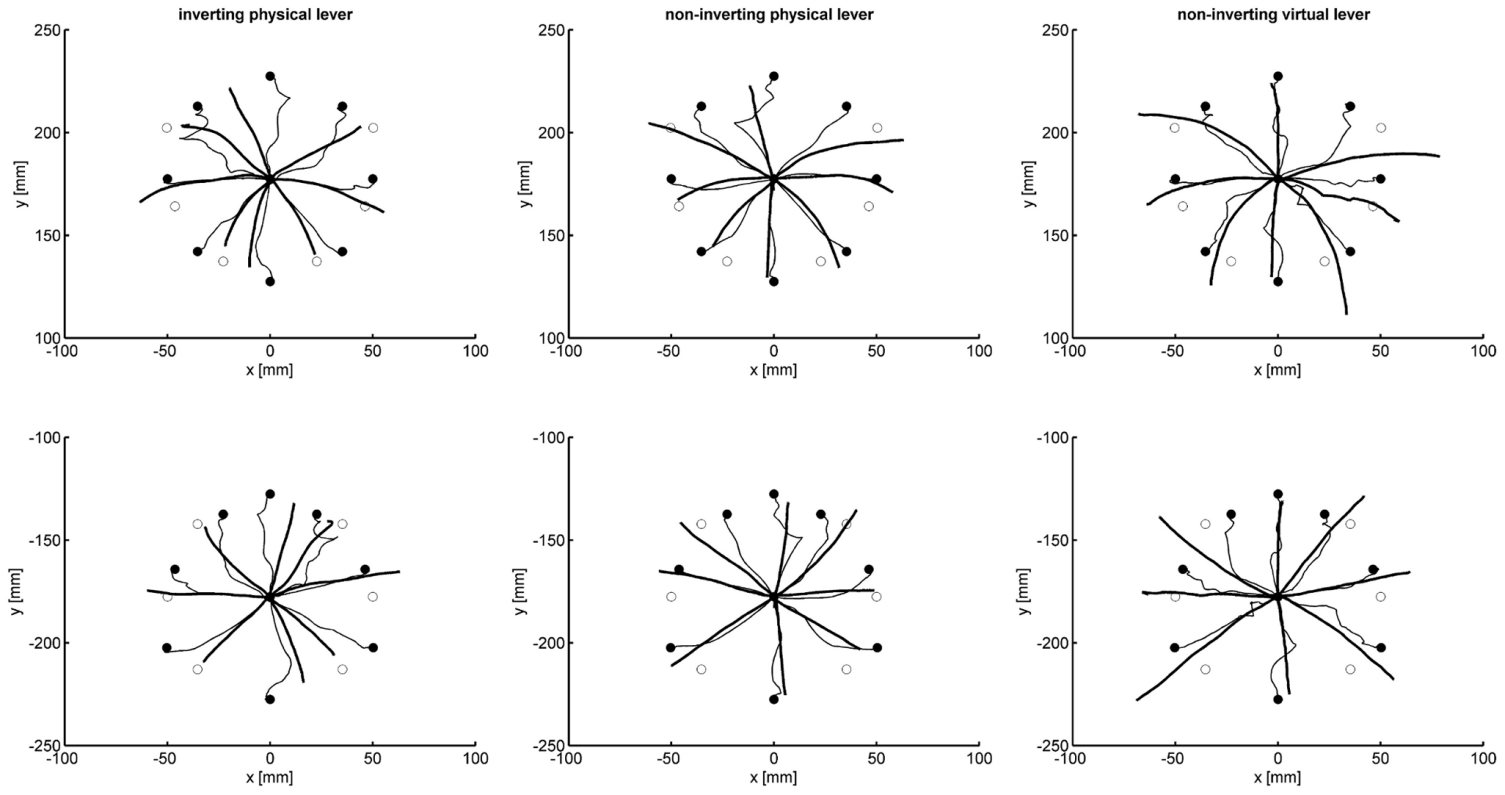
Mean cursor-motion paths (upper row of graphs) and mean hand-movement paths (lower row of graphs) of one participant of each experimental group in the first block with continuous (thin lines) and terminal (thick lines) feedback trials with the inverting physical lever. Filled circles mark correct endpoints, open circles endpoints appropriate for the symmetry approximation

Figure 4 presents averaged movement paths of one participant of each group in the first blocks of trials with the inverting physical lever with continuous visual-feedback (thin lines) and the subsequent block of trials with terminal visual-feedback (thick lines). The upper row of graphs shows the paths of the cursor, the lower row of graphs shows the paths of the hand. Of course, with terminal visual feedback the path of the cursor could not be perceived, and visual closed-loop control was impossible. The filled circles mark the positions of the targets presented on the monitor and the corresponding correct end positions of the hand movements. The open circles mark the end positions according to the line-symmetric approximation and the corresponding positions of the cursor.

Figure 4 illustrates some fundamental movement characteristics as they can be observed with a sliding first-order lever as a tool. First, with continuous visual feedback, movements were accurate at the end. Their initial direction, however, was more or less the same as that of the movements without continuous visual feedback to the same target. In-between, the averaged movement paths appeared somewhat erratic. This is a consequence of averaging quite diverse paths of individual movements. Second, in the absence of continuous visual feedback movement paths of the hand were rather straight, and the paths of the cursor were curved accordingly. The paths of the hand did not end at the correct positions, but generally close to the positions corresponding to the line-symmetric approximation.

### Practice

**Figure 5:**
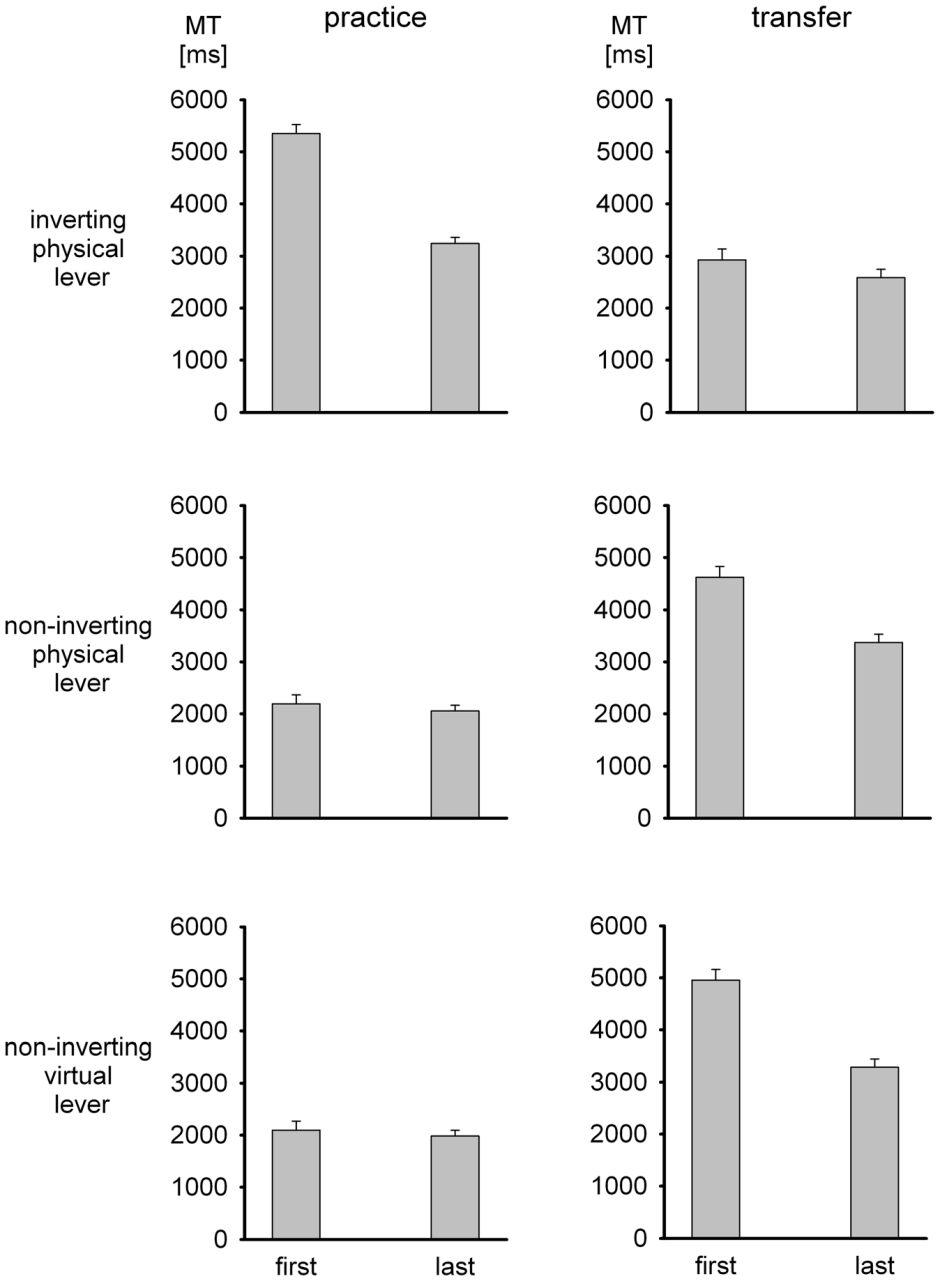
Mean movement times (with standard errors) of the three experimental groups during practice and transfer in blocks of trials with continuous visual feedback (first and last blocks of practice and transfer, respectively).

In the first and last practice block continuous visual feedback was presented. In these blocks accuracy was essentially perfect, but the time needed for the cursor to reach the target varied across groups and blocks of trials. Therefore movement time served as a performance criterion. Mean movement times in continuous-feedback trials of the practice phase are shown in Figure 5. Movement time was longer in group *inverting physical lever* than in the other two groups which hardly differed from each other. Whereas in group *inverting physical lever* there was a strong practice effect of 2111 ms, it was only small (137 and 115 ms) in the two groups who used a non-inverting physical or virtual lever. In spite of the different practice effects, a difference of 1224 ms remained at the end of practice. A two-way ANOVA with the between-participant factor group and the within-participant factor block of continuous-feedback trials (initial vs. final block of practice) revealed significant main effects of group, *F*(2,57) = 111.3, *p*<.001, and block, *F*(1,57) = 53.0, *p*<.001, as well as a significant interaction, *F*(2,57) = 51.5, *p*<.001.

**Figure 6:**
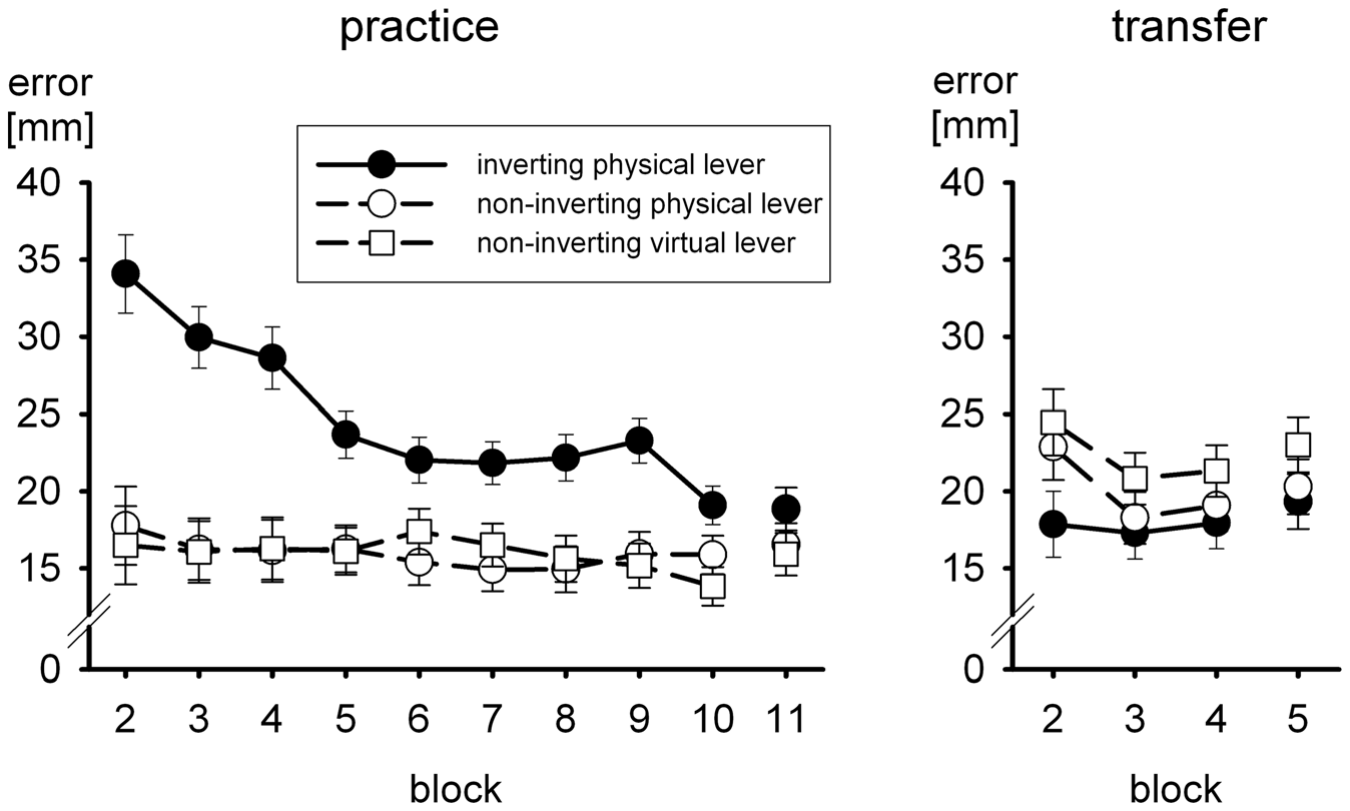
Mean Euclidean error (with standard errors) in trials without continuous visual feedback during practice and transfer. In practice block 11 and transfer block 5 there was no visual feedback at all, in the other blocks visual feedback was terminal

The first continuous-feedback block of practice was followed by nine blocks of trials with terminal visual feedback. For these trials accuracy of the cursor motion served as performance criterion. The mean errors are shown in Figure 6. For group *inverting physical lever* errors were larger than in the other two groups, which did hardly differ from each other. Whereas in group *inverting physical lever* there was a strong practice effect, with a difference of 15.0 mm between the first and last terminal-feedback practice block, the practice effect was only small (1.9 and 2.7 mm) in the two groups who used a non-inverting lever. At the end of practice a difference of 4.2 mm remained between the group with inverting lever and the two groups with non-inverting levers. A two-way ANOVA with the between-participant factor group and the within participant factor block (9 blocks) of terminal-feedback trials was run on the individual block means. The degrees of freedom were Greenhouse-Geisser corrected when appropriate, but we report the uncorrected degrees of freedom together with the Greenhouse-Geisser epsilon. Both the main effects of group, *F*(2,57) = 14.5, *p*<.001, and of block, *F*(8,456) = 9.5, *p*<.001, ε = .414, were significant, and so was the interaction, *F*(16,456) = 5.3, *p*<.001, ε = .414.

The nine terminal-feedback practice blocks were followed by a single block of trials without visual feedback. This block served to assess visual open-loop performance without any trial-to-trial guidance provided by terminal visual feedback that could result in inter-trial corrections. As is evident from Figure 6, the mean errors in the block without visual feedback (block 11) did hardly deviate from the errors in the final block of terminal-feedback trials (block 10). In fact, according to a two-way ANOVA with the between-participant factor group and the within-participant factor block (terminal feedback vs. no visual feedback) the slight increase from the last terminal-feedback block to the no-feedback block, 16.3 vs. 17.1 mm, was not significant, *F*(2,57) = 1.8, *p*>.10.

### Transfer

In the transfer phase all groups used the inverting physical lever. In the first and last blocks visual feedback was presented continuously during each movement. The mean movement times in these blocks are shown in Figure 5. For group *inverting physical lever*, who had used the same tool during practice, movement time continued to decline throughout the transfer phase. In contrast, for the two groups who had practiced with the non-inverting physical or virtual lever, movement time increased upon the change to the inverting lever. Throughout the transfer phase it declined again.

With respect to transfer, the main interest is in the initial performance with the inverting lever after practice with a non-inverting lever, in particular in its deviations from the initial performance with the inverting lever without preceding practice and performance with the inverting lever after practice with the inverting lever itself rather than the non-inverting lever. Movement times in groups *non-inverting physical lever* and *non-inverting virtual lever* were 4624 ms and 4954 ms, respectively, in the initial transfer block when they first used the inverting lever. Movement time of group *inverting physical lever* in the initial practice block, when this group first used the inverting lever, was longer, namely 5352 ms. Thus, with the preceding experience of a non-inverting lever movement time at the initial encounter with the inverting lever was faster, but not slower, than without such preceding practice. In other words, practice with a non-inverting lever resulted in positive and not in negative transfer to performance with an inverting lever. However, positive transfer was far from being perfect. With full transfer, movement time in the first transfer block after practice with a non-inverting lever should have been the same as after practice with an inverting lever. This was clearly not the case, with movement times of 4624 and 4954 ms as contrasted with 2927 ms.

For statistical analysis we compared the movement time in the first transfer block of groups *non-inverting physical lever* and *non-inverting virtual lever* with the movement times in the first practice and transfer blocks of group *inverting physical lever* by way of two one-way ANOVAs. For each ANOVA the three levels of the factor group were split into two contrasts. The contrast between the two groups who had practiced with the non-inverting lever was not significant in both analyses, indicating the lack of influence of the dynamic transformation. The contrast between group *inverting physical lever* (first practice block) and the other two groups (first transfer block) fell short of statistical significance, *F*(1,57) = 3.4, *p*<.10, indicating that the movement-time benefit associated with prior practice of the fine tuning was only marginally significant. For the analysis of the first transfer block of the three groups, the contrast was significant, *F*(1,57) = 80.7, *p*<.001, with the group who had practiced with the inverting lever from the very beginning moving faster than the two groups who had practiced the fine tuning only. Thus, transfer was clearly less than perfect.

The first block of trials with continuous visual feedback in the transfer phase was followed by three blocks of trials with terminal visual feedback and an additional block of no-feedback trials. For these trials accuracy of the cursor motion again served as performance criterion. The means are shown in Figure 6. In the two groups who had practiced with the non-inverting physical or virtual lever, there was a noticeable increase of the error in the first block of terminal-feedback trials that declined rapidly. With respect to transfer, the main interest is in the errors in the first terminal-feedback block of the transfer phase after the change of the tool in relation to the initial errors without any preceding practice and after practice with the inverting instead of the non-inverting lever. Mean errors in the first terminal-feedback transfer block were 22.9 and 24.5 mm in groups *non-inverting physical lever* and *non-inverting virtual lever*, respectively. The error of group *inverting physical lever* in its first terminal-feedback block of the practice phase was 34.1 mm. Thus, there was a clear positive transfer of practice with a non-inverting lever to performance with an inverting lever. However, transfer seemed to be less than perfect in that initial transfer performance after a change of the tool was somewhat poorer than the error of group *inverting physical lever* in the same block of trials (17.9 mm).

For the statistical analysis of transfer we ran two-way ANOVAs again. The comparison of the two groups who had practiced with the non-inverting lever was not significant in both analyses, confirming the lack of effect of the dynamic transformation also in open-loop trials. However, the contrast of initial practice performance of group *inverting physical lever* with initial transfer performance of the two other groups was significant, *F*(1,57) = 7.4, *p*<.01. Prior practice of the fine tuning therefore resulted in a significant increase of accuracy with the inverting physical lever as compared to no prior practice, that is, there was a reliable positive transfer. The contrast of the initial transfer performance of group *inverting physical lever* with initial transfer performance of the two other groups was significant as well, *F*(1,57) = 4.9, *p*<.05, indicating that prior practice of the inverting physical lever led to more accurate movements as compared to the practice of only the fine tuning. Thus, positive transfer was present, but it was less than perfect.

### Left-right inversion and fine tuning

**Figure 7:**
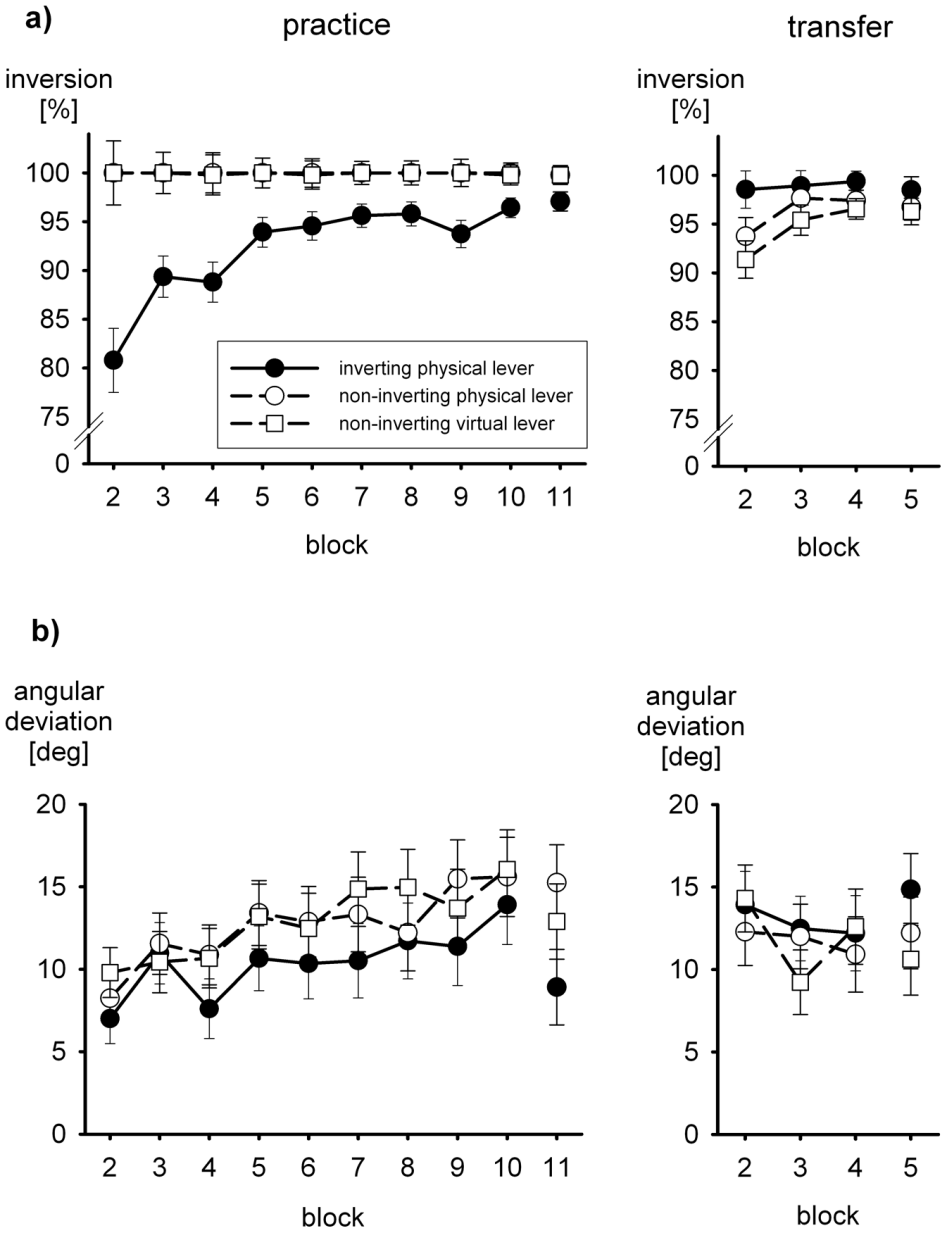
Mean percentage of movements with right-left inversion (a) and mean angular deviation (b) during practice and transfer. Error bars represent standard errors.

Errors in visual open-loop trials without continuous visual feedback result from imperfections of the right-left inversion and the fine tuning. For each block of terminal-feedback trials and no-feedback trials the quality of inversion was assessed in terms of the percentage of hand movements that ended in the quadrant around the correct final hand position, that is, by the percentage of hand movements with a directional error of less than 45°. The mean percentages are shown in Figure 7a. Except for very few blocks of trials, with the non-inverting lever all movements of all participants ended in the correct quadrant. A statistical analysis of these data is not meaningful because of the absence of variability. However, with the inverting lever there was variability, and a certain proportion of hand movements did not end in the correct quadrant. In group *inverting physical lever* there was improvement during practice. In the other two groups the percentage of hand movements that ended in the correct quadrant declined abruptly when the tool was switched to the inverting lever at the start of the transfer phase; in the subsequent blocks of the transfer phase there was a rapid improvement.

In the first block of terminal-feedback trials in the transfer phase, that is, after the shift from the non-inverting lever to the inverting lever, the mean percentages of hand movements that ended in the correct quadrant were 93.7 and 91.4% in groups *non-inverting physical lever* and *non-inverting virtual lever*, respectively. This compares with a percentage of 80.8% at the start of the practice phase and 98.5% at the start of the transfer phase in group *inverting physical lever*. Thus, practice with a non-inverting lever resulted in positive transfer to performance with an inverting lever as far as the quality of right-left inversion was concerned, but positive transfer was less than perfect.

For the statistical analysis we ran again two one-way ANOVAs to compare the initial transfer performance in the two groups with a tool switch to initial practice and transfer performance in the group who had practiced with the inverting lever from the very start. The three levels of the factor group were again split into two contrasts. In both analyses the initial transfer performance of groups *non-inverting physical lever* and *non-inverting virtual lever* was not significantly different. However, initial transfer performance of these two groups was significantly better than initial practice performance of group *inverting physical lever*, *F*(1,57) = 6.4, *p*<.05, confirming positive transfer. With respect to initial transfer performance of group *inverting physical lever*, the difference to initial transfer performance of the other two groups was also significant, *F*(1,57) = 6.5, *p*<.05, confirming that positive transfer was less than perfect.

The quality of the fine tuning for those hand movements that ended in the correct quadrant was assessed in terms of the mean deviations of angles between pairs of movement directions from those expected for a perfect line-symmetric approximation (see Figure 3). Hand movements to the correct end positions would result in deviations of 33.4°. The mean angular deviations are shown in Figure 7b. There was a slow improvement during practice, with only little or no differences between groups. For the statistical analysis we subjected the individual mean angular deviations to a two-way ANOVA with the factors group and block (9 blocks of terminal-feedback trials). The main effect of block was significant, *F*(8,456) = 7.8, *p*<.001, ε = .520, whereas the main effect of group and the interaction were not, *F*<1. In a second ANOVA we tested the potential role of terminal visual feedback for the maintenance of the fine tuning. Only the last practice block with terminal visual feedback (block 10) and the subsequent block without visual feedback (block 11) were included. In this ANOVA the factor block turned out to be significant, *F*(1,57) = 10.5, *p*<.01, but the interaction with group fell short of statistical significance, *F*(2,57) = 2.4, *p*>.10, and so did the main effect of group.

For the assessment of transfer of the fine tuning it is important that fine tuning was acquired equally with the different types of tool studied. Therefore, evidence of positive transfer would be revealed by a better fine tuning at the start of the transfer phase than at the start of practice. Less than perfect transfer would be indicated by a poorer fine tuning in the first transfer block after the tool switch in groups *non-inverting physical lever* and *non-inverting virtual lever* than in group *inverting physical lever*, in which there was no tool switch. For the statistical analysis we entered the first practice block and the first transfer block in an ANOVA. Only the main effect of block was significant, *F*(1,57) = 17.0, *p*<.001, indicating positive transfer. The three-level factor group was again split into two contrasts. None of these contrasts or of the interactions with block approached statistical significance, all *F*<1. Thus, transfer was indistinguishable from being perfect.

## Discussion

The purpose of the present study was to investigate the effects of part-task training on the acquisition of an internal model of a complex visuomotor transformation. Part-task training is a well-established procedure, in particular for difficult tasks for which simplification results in levels of difficulty that are better suited to optimize learning and motivation [28]-[29]. It should be particularly efficient for skills that can be decomposed in components that are learned independently [30]–[31]. Here we use the efficiency of part-task training as evidence of distinct adaptive processes with different time scales.

We simplified the complex transformation of a sliding first-order lever according to two hypothesized processes [10]–[12], a rapid line-symmetric approximation (or right-left inversion) and a slow graded fine tuning. In terms of overall performance, part-task training of the fine tuning resulted in positive, but less than full transfer both in visual closed-loop and open-loop trials. This is to be expected because part-task training was limited to only one of the hypothesized processes, namely the fine tuning. According to a dependent variable which served to tap the quality of fine tuning, however, positive transfer for this process was indistinguishable from being perfect. Somewhat unexpectedly, the part-task training of the fine tuning produced also positive transfer on the quality of right-left inversion, even though the levers used during part-task training were non-inverting.

The conclusion that positive transfer of the fine tuning acquired with a non-inverting lever to the fine tuning with an inverting lever is essentially perfect should be accepted with some reservation only for statistical reasons. Our analyses of transfer of practice with a non-inverting lever were based on comparisons of initial transfer performance with an inverting lever with two reference performance levels. Initial performance without preceding practice was the reference for absence of positive transfer, and performance after an equivalent amount of practice with the inverting lever was the reference for perfect transfer. Statistically significant differences from the reference levels indicate positive transfer and incompleteness of positive transfer, respectively. If only one of the comparisons is significant, there is positive transfer that might be perfect or incomplete transfer that might even be absent. If none of the comparisons is significant, no conclusion is possible. For the transfer of fine tuning, thus, positive transfer is clearly present and – in a strict statistical sense – the hypothesis of perfect transfer cannot be rejected. Note that a reasonable test of perfect transfer is impossible because the alternative effect size is arbitrarily small so that statistical power is arbitrarily small as well. However, as far as the transfer of fine tuning is concerned, the existence of positive transfer is sufficient to show that it is acquired in the absence of the right-left inversion and transferred to its presence. Thus, the statistical reservations about the full transfer are not essential for the current conclusions.

Our findings provide further evidence of distinct components of internal models of motor transformations. As such they are in line with previous studies which showed compositions of previously learned components [24]–[26], [32]. These findings support the general proposition that the “divide-and-conquer” approach associated with the part-task training is congruent with a modular computational strategy underlying motor learning, e.g., the so-called “mixture of experts” model (cf. [33]).

Beyond the general addition to previous results, our findings provide more specific evidence of distinct processes of visuo-motor adaptation to complex transformations with different time scales – rapid and slow – and different outcomes – discrete and graded changes of movement directions. We show that the slow process with graded outcomes can be separated from the fast process with discrete outcomes, and that the rapid process can be added later on. Thus, the natural order of the processes can be reversed in that the outcome of the slow process becomes available earlier than the outcome of the fast process.

Our observations were made with a specific kinematic transformation, that of a sliding first-order lever. However, they might also apply to other transformations for which rapid processes with discrete outcomes and slow processes with graded outcomes play a role. Such transformations are large visuomotor rotations between 90° and 180° [6] and right-left reversals [7]. According to the present results, the different processes produce modular outcomes that can be composed irrespective of their temporal order. The different types of adaptive process might be related to discrete and graded specification of movement characteristics, in particular movement directions. For example, discrete and graded specifications of new movement directions have been reported for large and small changes of direction, respectively [34].

The part-task training of the fine tuning not only transferred to the fine tuning when the inverting lever was introduced, but also resulted in more correct inversions as compared to no prior practice of any feature of the lever transformation. At present we can only speculate on the origin of this somewhat unexpected observation. One way to explain these unspecific transfer effects is that the exposition to any kind of novel visuomotor transformation improves the participants' ability to adapt in general. This “learning to learn” mechanism has previously been associated with performance benefits in the adaptation to discordant visuo-motor transformations [35]. An alternative explanation is related to the fact that the concurrent rapid and slow processes of adaptation, even when they are functionally independent, are driven by a single movement error. The problem of decomposing the error into components that drive the concurrent processes of adaptation more or less disappears when one process has reached asymptote. Thus, after part-task practice of the fine tuning, the remaining error could be more accurately ascribed to the inversion.

In the present study we not only separated two characteristics of the kinematic transformation of the sliding first-order lever, but in addition we separated the kinematic transformation from the dynamic one. There were no differences at all between the two groups who had practiced with a physical and a virtual non-inverting lever. This observation supports the notion that the acquisition of an internal model of the kinematic transformation of a sliding first-order lever depends only marginally or not at all on the presence or absence of the dynamic transformation [10]-[11] even though both are mechanically related to each other. In addition, the absence of the dynamic transformation changes the relations between forces exerted by the hand and movements of the tip of the lever. The lack of effect of the dynamic transformation of a sliding lever on movements of the hand has recently been attributed to the fact that the rather small and complex variations of inertia are compensated by an increased impedance of the limb [36].

The present evidence of distinct adaptive processes with outcomes that can be combined independently of their temporal order strongly suggests that these processes can also be dissociated in terms of their neural substrates. Even though the precise neural substrates of the fast and slow processes are not yet fully clear, some findings support the assumption of distinct neural structures [37], with the cerebellar cortex being associated with a fast process and the cerebellar nuclei with the slow process. Further evidence for distinct representations comes from a study linking only the fast, but not the slow process to declarative memory [38], whereas the slow process has been associated with procedural memory [39]. It has also been shown that especially the fast process engages spatial working memory, with associated activity in the right dorsolateral prefrontal cortex and the bilateral inferior parietal lobes [40]-[41].
